# Quinces (*Cydonia oblonga*, *Chaenomeles* sp., and *Pseudocydonia sinensis*) as Medicinal Fruits of the Rosaceae Family: Current State of Knowledge on Properties and Use

**DOI:** 10.3390/antiox13010071

**Published:** 2024-01-03

**Authors:** Anna Kostecka-Gugała

**Affiliations:** Department of Plant Biology and Biotechnology, Faculty of Biotechnology and Horticulture, University of Agriculture in Krakow, al. Mickiewicza 21, 31-120 Kraków, Poland; anna.kostecka-gugala@urk.edu.pl

**Keywords:** polyphenols, antioxidant, functional food, anticancer, antidiabetic

## Abstract

In recent years, the evaluation of many plant-derived compounds as potential new drugs or functional foods has become an active research topic. The morphological characteristics of quinces of the genera *Cydonia* sp., *Chaenomeles* sp., and *Pseuocydonia* sp. are largely similar, which is why these fruits are often confused. Although they have been appreciated in Asia for centuries as a valuable component of local ethnomedicine, they are less known in Western countries, and scientific knowledge about their health benefits remains fragmentary. This literature review summarizes studies on the content of chemical compounds responsible for the health-promoting and functional properties of the quince fruit. It focuses on the content of carotenoids, vitamins, minerals, and carboxylic acids, although the main emphasis is on the content and diversity of bioactive polyphenols, which are extremely abundant in these fruits. The quince fruits are rich in antioxidants and compounds with proven anti-inflammatory, anticancer, antiallergic, and immunomodulatory effects. Their phytochemicals effectively regulate glycemia and improve the blood lipid profile, suggesting potential antidiabetic and cardioprotective benefits. Analysis of chemical characteristics showed that the *Chaenomeles* fruits. are underestimated as functional food ingredients. Studies on the molecular effects of their bioactive compounds and species-specific genomic analyses are sorely lacking in the scientific literature.

## 1. Introduction

For the consumer, the fruits of *Cydonia oblonga* Mill., *Pseudocydonia sinensis* (Thouin) Schneid., and *Chaenomeles* sp. Lindl. are similar: they are yellow, hard, fragrant, have a sour taste, and are used in similar ways. For growers and gardeners, these are plants with different purposes (consumption, medicinal, ornamental) and different requirements. In some languages, there are no separate names for the fruits of *Cydonia*, *Pseudocydonia,* or *Chaenomeles*. In English, adjectives are sometimes added, such as Turkish, Chinese, Japanese, Persian, etc. Not surprisingly, their fruits are confused even in the professional and scientific literature [1]. Distinguishing the fruits is further complicated by the number of Latin synonyms. The relative ease of obtaining hybrid forms within the genus *Chaenomeles* results in additional difficulties in identification.

The aim of this work was to present the chemical composition of their fruits and a large number of medical applications as well as to improve the distinguishability of the various quince taxa, especially in Western countries where these plants are still poorly recognized. Due to the vastness of the subject, the article omits detailed aspects of biology, cultivation, or culinary use and, in describing the composition, focuses mainly on the polyphenolic compounds in which the fruits of these species are rich, and which are largely responsible for their various health-promoting properties.

## 2. Characteristics of Quince Plants, Their Biology, Cultivation and Culinary Use

The plant Cydonia oblonga Mill. originates from Western Asia, more precisely from the Transcaucasian region [2]. The species is widespread due to its cultivated forms and is naturalized in the area of the Mediterranean Sea. It is a monotypic genus of the family Rosaceae.

Species of the genus *Chaenomeles* have been known in China for thousands of years, and their fruits are used in traditional Chinese medicine (TCM). Interest in the fruits has increased in recent decades due to the possibility of cultivating several species of these plants in Europe, mainly in the Baltic countries [3]. The genus *Chaenomeles*, a member of the Rosaceae family, consists of four species, namely *Chaenomeles speciosa* (Sweet) Nakai, *Chaenomeles thibetica* Yü, *Chaenomeles cathayensis* Schneid., and *Chaenomeles japonica* (Thunb.) Lindl. ex Spach., which are naturally distributed in eastern Asia [4]. These taxa can easily interbreed, both spontaneously and as a result of deliberate hybridization—More than 500 varieties with ornamental flowers have been described in the literature; therefore, *Chaenomeles* shrubs are sometimes called “flowering quince”. Another member of the Rosaceae family is *Pseudocydonia sinensis* Schneid., the only species of the genus *Pseudocydonia*. Its taxonomic status has changed over time and remains controversial [5]. This species was placed in the genus *Cydonia* and later in the genus *Chaenomeles* (*C. sinensis* Kochne). Recent advanced and comprehensive LM and SEM studies of pollen morphology confirmed the placement of this species in the monotypic genus *Pseudocydonia* [5]. This species is native to southern and eastern China but has also been introduced to Japan, Korea, and the USA.

Many taxonomists emphasize that the genera *Chaenomeles* and *Cydonia* are closely related, but in the genus *Chaenomeles,* many hybrids have been described, while in the genus *Cydonia* hybridization can only occur within *C. oblonga* and *P. sinensis* [6].

**Figure 1:**
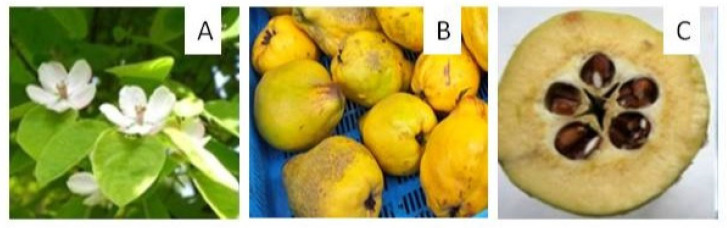
Flowers and fruits of *Cydonia oblonga* Mill. Source: photo by Monika Bieniasz, Univ. of Agriculture in Kraków, Poland (**A**,**C**); photo by the author (**B**).

*C. oblonga* is a shrub or, more commonly, a small tree. Its flowers are white or pink, large and solitary, and the fruits are spherical to oblong (8–12 cm in diameter), with an average weight of 100–250 g (Figure 1). The color of the epidermis changes from brown to light green in the early stages of development and turns yellow when ripe [2]. The flesh is yellowish, consistent, slightly sweet, often astringent. Due to the high content of organic acids, the pulp of most cultivars is not eaten raw but in the form of jams, marmalades, juices, jellies, puddings, compotes, and cakes [7,8,9,10,11,12]. The naturally dried fruits of *Cydonia* have been used as tea in Asia Minor for centuries [13]. A unique product from the Iberian Peninsula named “quince cheese”, is a reddish, hard, sticky, and sweet paste. The use of *C. oblonga* fruits as an ingredient of alcohols is also widespread, including for the production of a brandy-type “rakija”, famous in the Balkans, or as an ingredient in other alcoholic beverages [14]. Attempts have also been made to improve beer with a macerate of *Cydonia* fruit, which enhances its sensory qualities [15].

**Figure 2:**
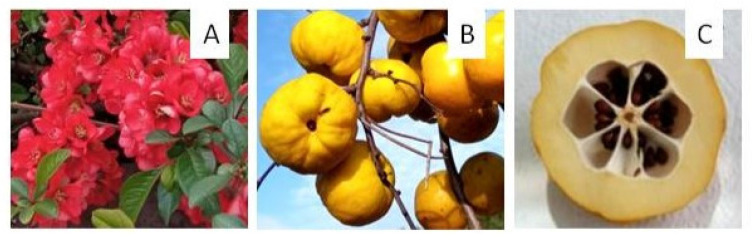
Flowers and fruits of *Chaenomeles japonica* (Thunb.) Lindl. ex Spach. Source: photo by the author (**A**,**B**); Monika Bieniasz, Univ. of Agriculture in Kraków, Poland (**C**).

Plants of the *Chaenomeles* genus are shrubs, usually growing to 2–3 m in height; only *C. cathayensis* grows as a small tree up to 6 m tall, while *C. japonica* is usually shorter. *Chaenomeles* flowers have unique decorative value; they appear before the leaves unfold and may be white, pink, orange, or red (Figure 2). The fruits are smaller, up to 100 g, spherical, and very sour (a titratable acidity of 47.5% malic eq. was measured for *C. japonica*) [16], which makes them unsuitable for raw consumption. However, due to their unique sensory properties, including intense aroma, they are used as additives that can enrich other products with valuable properties, e.g., teas, yogurts, cold drinks, liqueurs, ice cream, cocktails, and cottage cheese [17,18,19]. They are highly valued as dried candied fruits [20,21]. Freeze-dried fruits were added to cookies in order to improve their volatile characteristics and acceptability by consumers and to maintain quality during storage due to the strong antioxidant properties of the fruits [22]. Nawirska-Olszańska et al. [17,23] analyzed the properties of pumpkin jam mixed with *C. japonica* fruits and proved that such an additive enriched it with phenolic compounds and ascorbic acid as well as improved the volatile profile more significantly than using other fruits for this purpose.

**Figure 3:**
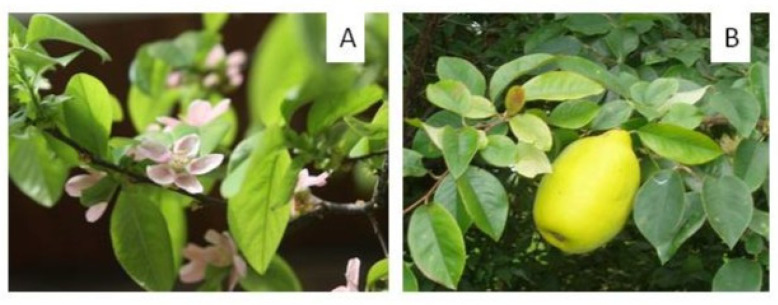
Flowers and fruit of Psudocydonia sinensis Schneid. Source: photo, Dalgial, CC BY-SA 3.0 (https://creativecommons.org/licenses/by-sa/3.0, accessed on 21 December 2023), via Wikimedia Commons (**A**) and Tusbra (public domain, Wikimedia Commons) (**B**).

*Psudocydonia sinensis* is an attractive ornamental tree growing up to 18 m high. What distinguishes it from the genus *Chaenomeles* is the lack of thorns, single, unclustered flowers (Figure 3), and the exfoliating bark. The flowers are decorative and appear later than in most *Chaenomeles* shrubs. The fruit itself resembles *Cydonia*; it is an ovoid pome 12–17 cm long but without tufts. It is also hard and astringent, but after frost, these properties weaken. Nevertheless, the fruit is considered unpalatable due to the high lignin content (24.5% dm) [24]. Therefore, it is processed into jams, syrups, liqueurs, wine, and jellies [25,26].

In the culinary and pharmaceutical uses of *Cydonia*, *Chaemnomeles*, and *Pseudocydonia*, an important point to keep in mind is the content of cyanogenic glycosides (mainly amygdalin) in their seeds. The potential toxicity of amygdalin results from enzymatic degradation in the human digestive system leading to the production of toxic HCN [27]. However, according to the literature, *C. oblonga* seeds contain low levels of amygdalin, making this fruit suitable for new applications in food technology and functional food design [28,29]. Detailed studies have been carried out on *C. japonica* seeds. Mierina et al. [30,31] determined the HCN content to be at the level of 0.3 and 0.7 mg/g in seeds. This concentration is similar to that found in apple seeds, which can range from 0.9 to 3.9 mg/g of seed [32]. Therefore, direct consumption of *Chaenomeles* seeds without pretreatment may be hazardous to human health. On the other hand, tests on cold-pressed oil did not show any amygdalin content [33]. Analyses of different fractions obtained from pressed *C. japonica* seed residues revealed the absence of amygdalin in all oil extracts obtained with or without ethanol as a co-solvent. Amygdalin was extracted together with polyphenols at levels up to 118 μg/mL, corresponding to 3080 μg/g dm of defatted *C. japonica* residue. In contrast, protein isolates obtained with or without tannin removal were free of amygdalin [34].

## 3. Chemical Composition of Quince Fruits and Seeds

### 3.1. Phenolic Compounds

Many reports show the content of phenolic phytochemicals in *Cydonia* fruits: in their pulp [35,36,37,38,39,40], peel [35,36,39,40,41,42], and seeds [38,41,43]. A few papers focused on beneficial compounds in leaves [39,44,45,46,47,48]. Some authors used whole fruits for analysis [49,50] or their callus [51]. The data on the content of polyphenols in fresh and dried pulp, peel, and seeds are summarized in Table 1, Table 2 and Table 3.

Importantly, *C. oblonga* fruit contained approximately twice as much total phenolics as apple fruit, both of which are used as raw materials [56]. All *Cydonia* organs contained significant amounts of phenolic acids; especially neochlorogenic (3-*O*-caffeoylquinic), cryptochlorogenic (4-*O*-caffeoylquinic), and chlorogenic (5-*O*-caffeoylquinic) acids have been widely reported. Since the numbering of chlorogenic acid atoms remains ambiguous, this review follows the nomenclature adopted by the authors of the cited papers.

Research by Andrade et al. [52] showed that the pulp contained significantly more chlorogenic acid (6770 mg/kg) than the closely related apples and pears. Sut et al. [40] showed 411 mg/kg chlorogenic acid in the peel, which was significantly less than in the pulp, while in several apple cultivars, the amounts ranged from 5 to 305 mg/kg. Typical *Cydonia* flavonoids were kaempferol, quercetin, and their various glycosidic derivatives; however, they appeared to be less abundant components compared to procyanidins and chlorogenic acid derivatives [40,57]. Analysis of flavonoid content showed that quince is a rich source of quercetin-*O*-3-galactoside, quercetin-*O*-3-rhamnoside, and quercetin-*O*-3-rutinoside (rutin) compared to their content in apple and pear pulp. In the case of rutin, about four times more was found in the pulp than in apple pulp [52].

While the content of phenolic acids and flavonoids in various quince tissues has been relatively well documented, there are few data on the content of tannins. Based on the data presented by Sharma et al. [58], we know that they are found in fruit juice at a level of 0.8%. The content of procyanidin B_1_ in the pulp was significantly higher (65 mg/kg) than in the pulp of apple fruits (2–19 mg/kg).

In general, a higher content of bioactive compounds was found in the peel of *C. oblonga* than in the pulp [37]. Both pulp and peel contained large amounts of caffeoylquinic acids, mainly types 3- and 5-, but the peel turned out to be a reservoir of flavonoid compounds. However, the differences in their amounts reported in the literature are significant. The final result of phenol content depends on the choice of cultivar, growing conditions, including soil pH, humus, and mineral content, length of the growing season, weather conditions, and the overall physiological state of plants. In addition, the fruit ripeness, duration, and storage conditions are very important. Quinces are known for being able to be stored for a long time, but the storage process affects the content of individual bioactive compounds. Each research group individually selects the extractant, which has a significant impact on the number and proportions of extracted compounds. In addition, variations in drying and extraction methods, as well as differences in measurement techniques, account for the differences observed in Table 1, Table 2, Table 3 and Table 4. According to research on the extraction of *C. japonica* phenolics, the most effective extractants among the nine tested were 50% ethanol, 100% methanol, and 50–70% acetone [59].

*C. oblonga* seeds, in turn, contained significantly fewer phenolics in total, but their more considerable diversity was observed. Among them, less common compounds could be distinguished, such as flavonoid di-*C*-glycosides: schaftoside, isoschaftoside, lucenin-2, stellarin-2, and vicenin-2 [36,38,41].

One research article is particularly important in that it compares the polyphenol content in the fruits (and leaves) of *C. oblonga*, *C. japonica*, and apple [47]. The authors identified 2909 mg of phenolic compounds in *C. oblonga*, as much as 7643 mg in *C. japonica*, while apples contained only 1312 mg per 100 g of dry mass (dm), which means that *C. oblonga* fruits were twice as rich in phenolics as apples and, which is worth emphasizing, *C. japonica* fruits were about three times richer in phenolic compounds than the *Cydonia* fruits.

The analysis of leaf phenolics revealed that they were more abundant in the leaves of *C. oblonga* than in the leaves of other species known for their high phenolic content, such as chokeberry, cranberry, blueberry, and blackcurrant. However, the more significant differences in the content of specific phenolics were observed in the case of mono-, di-, and oligomeric flavan-3-ols; *C. japonica* fruits contained 4595 mg/100 g dm, while *C. oblonga* fruits contained more than 50 times less. In turn, both the fruits and the leaves of *C. oblonga* were about three times richer in phenolic acids (273 and 3894 mg/100 g dm, respectively) than the fruits and leaves of *C. japonica*. The contents of flavonols in the leaves and fruits of the two compared quince species were similar [47].

Unfortunately, we have much less data on the polyphenol contents of both fresh and dried *Chaenomeles* and *P. sinensis* fruits (Table 4). The analysis is complicated by the fact that sometimes extracts were prepared from whole fruits after removal of the seed core, probably due to the smaller size of the fruits, and then freeze-dried, in which case the results of all measurements were expressed on a dry weight basis, while other researchers used extracts from fresh pulp. Urbanavičiūtė et al. [59] analyzed the total phenolic content in *C. japonica* extracts and found that it ranged from 4523 to 6785 mg/100 g dm. Significant differences in the obtained values resulted from specific combinations of parameters (i.e., type of solvent, time, power, and temperature of ultrasonic extraction). Tarko et al. [60], reported that the total phenolic content was 924 mg catechin equivalents per 100 g dm, which is approximately50% higher than that found in the fruits of cornelian cherry and black mulberry, both of which are known for their high phenolic content. Several studies identify numerous phenolic compounds in these fruits [3,28,47,61,62,63,64], but only a few of them provide numerical values. Among those already mentioned, there are phytochemical studies that showed the presence of flavonoids [63,65], lignan glycosides [66], biphenyl derivatives [67], as well as essential oils [68], pentacyclic triterpenes [62,63] and sesquiterpenoids [62], the last three outside the polyphenol group. In turn, in the group of *C. japonica* flavonols, those that were highly abundant in fruits and leaves were indicated, i.e., (+)-catechin, (–)-epicatechin, and procyanidins B_1_, B_2_, B_3_, and C_1_ [47]. Among the bioactive compounds, polyphenols (mainly phenolic acids and flavonoids) and triterpenes were considered to be the major classes of phytochemicals in *C. speciosa* [4].

An interesting study was carried out by Hellín et al. [69], who used fruit juice from five taxa of the genus *Chaenomeles* (*C. japonica*, *C. speciosa*, *C. cathayensis*, *C. japonica* × *C. speciosa*, and *C. × superba*) and determined 210–592 mg of phenolic compounds in 100 mL of juice obtained from *C. japonica* fruits collected from different locations. Juices of other *Chaenomeles* species contained even more phenolics, i.e., 591 mg in 100 mL for *C. cathayensis*. These amounts were significantly higher than in apple juice (339 mg/100 mL) [70]. Du et al. [62] presented a comparison of the amounts of major phenolic compounds in fruits of five *Chaenomeles* species. They showed an abundance of chlorogenic acid in *C. speciosa*, *C. thibetica,* and *C. cathayensis* and a low content in *P. sinensis* and *C. japonica.* Catechin and procyanidin B_1_ were abundant in *C. thibetica* and *C. cathayensis* and moderate in *C. speciosa*. On the contrary, epicatechin and procyanidin B_2_ were predominant in *C. speciosa*, *P. sinensis,* and *C. japonica*. Research by Vila et al. [71] confirmed that *Chaenomeles* fruits from southern growing areas contained significantly more phenolic compounds than those from northern growing areas. During ripening, the total phenolic content showed a slight tendency to decrease from two weeks before harvest. This pattern is consistent with observations made for many Rosaceae fruits.

**Table 4:** Phenolic contents in *Chaenomeles* sp. and *Pseudocydonia sinensis* fresh and dried fruits (extracted with water:acetone).

Compound	Species	Content		Ref.
Apigenin	*C. japonica*	19.66	mg/100 g dm	[28]
3-CQA (5-*O*-caffeoylquinic acid) neochlorogenic acid	*P. sinensis*	5.00	mg/100 g fm	[53]
4-CQA (5-*O*-caffeoylquinic acid) cryptochlorogenic acid	*P. sinensis*	1.20	mg/100 g fm	[53]
5-CQA (5-*O*-caffeoylquinic acid) chlorogenic acid	*C. japonica*	818.55	mg/100 g dm	[28]
		10.00	mg/100 g fm **	[62]
		12.17	mg/100 g dm *	[72]
	*C. speciosa*	182.00	mg/100 g fm **	[62]
	*C. thiberica*	117.00	mg/100 g fm **	[62]
	*C. cathayensis*	119.00	mg/100 g fm **	[62]
	*P. sinensis*	9.00	mg/100 g fm **	[62]
		0.50	mg/100 g fm	[53]
(+)-Catechin	*C. japonica*	15.75	mg/100 g dm *	[72]
Catechin	*C. japonica*	121.12	mg/100 g dm	[28]
	*C. speciosa*	54.00	mg/100 g fm **	[62]
	*C. thiberica*	156.00	mg/100 g fm **	[62]
	*C. cathayensis*	113.00	mg/100 g fm **	[62]
	*P. sinensis*	5.00	mg/100 g fm **	[62]
		2.90	mg/100 g fm	[53]
*trans*-Cinnamic acid	*C. japonica*	18.72	mg/100 g dm	[28]
*p*-Coumaric acid	*C. japonica*	5.72	mg/100 g dm	[28]
(–)-Epicatechin	*C. japonica*	348.44	mg/100 g dm *	[72]
Epicatechin	*C. japonica*	102.00	mg/100 g fm **	[62]
	*C. speciosa*	235.00	mg/100 g fm **	[62]
	*P. sinensis*	54.00	mg/100 g fm **	[62]
		11.90	mg/100 g fm	[53]
2,5-di-Hydroxybenzoic acid	*C. japonica*	2.01	mg/100 g dm	[28]
4-Hyrdoxybenzoic acid	*C. japonica*	1.92	mg/100 g dm	[28]
Ferulic acid	*C. japonica*	2.17	mg/100 g dm	[28]
Isoquercitrin	*C. japonica*	3.82	mg/100 g dm	[72]
Naringenin	*C. japonica*	5.92	mg/100 g dm	[28]
Procyanidin B_1_	*C. speciosa*	83.00	mg/100 g fm **	[62]
	*C. thiberica*	222.00	mg/100 g fm **	[62]
	*C. cathayensis*	145.00	mg/100 g fm **	[62]
	*P. sinensis*	13.00	mg/100 g fm **	[62]
		9.80	mg/100 g fm	[53]
Procyanidin B_2_	*C. japonica*	98.00	mg/100 g dm **	[62]
	*C. speciosa*	296.00	mg/100 g fm **	[62]
	*P. sinensis*	16.80	mg/100 g fm	[53]
		40.00	mg/100 g fm **	[62]
Quercitin	*C. japonica*	5.03	mg/100 g dm	[28]
Q-3-R (quercetin-3-*O*-rutinoside) rutin	*C. japonica*	107.09	mg/100 g dm	[28]
		5.40	mg/100 g dm *	[72]
Sinapic acid	*C. japonica*	27.99	mg/100 g dm *	[72]
Syringic acid	*C. japonica*	0.03	mg/100 g dm	[28]
Vanilic acid	*C. japonica*	13.69	mg/100 g dm	[28]

fm—fresh mass, dm—dry mass. * Results for water: ethanol extract. ** Results for the pulp.

The seed sockets of *C. japonica* fruits are large compared to the size of the mesocarp. It is therefore not surprising that attempts have been made to use the seeds. Dried seeds contain approximately 6–16% oil [73]. Cold pressing resulted in an oil with promising health-promoting properties. It contained the highest amount of polyphenols (64 mg/kg) compared to sesame, poppy, peanut, flaxseed, pumpkin, sunflower, almond, hazelnut, and walnut oils [34]. Six phenolic compounds were found in it, viz., 4-hydroxybenzoic acid, vanillic acid, vanillin, *p*-coumaric acid, ferulic acid, and *trans*-cinnamic acid [74]. In turn, Turkiewicz et al. [75] studied the content of essential phytochemicals in *Chaenomeles* leaves and concluded that they could be a good material for obtaining extracts rich in phenolics, mainly procyanidins, and quercetin and its glycosides.

Our understanding of the polyphenolic components of *P. sinensis* fruit is limited. The phenolic total content, which was measured via the Folin–Ciocalteu assay, was found to be 1280 mg/100 g of fresh mass (fm). It was about four times higher than that of *C. oblonga* fresh fruit (303 mg/100 g) and 20 times higher than that of apple fresh fruit (61 mg/100 g) [53]. A more detailed study showed that *P. sinensis* fruit contained 24 phenolic compounds, of which 20 were flavan-3-ols such as catechin, epicatechin, and procyanidins, which accounted for 94–99% of the total polyphenols [53,76]. Research by Hamauzu and colleagues [25] showed the presence of polyphenols in the aqueous solution of *P. sinensis*, including procyanidin B_3_, (+)-catechin, procyanidin B_4_, procyanidin B_2_, (–)-epicatechin, and oligomeric and polymeric procyanidins. It is worth emphasizing that procyanidins are compounds found in the fruits of all described genera, with a great diversity of them found in the fruits of *Chaenomeles* sp. and *P. sinensis*. However, because the fruits of *C. oblonga* are generally the best known, procyanidins are often associated with them. As shown, the content of polymeric procyanidins decreased during heat treatment. Changing the ratio of polymeric to oligomeric and monomeric forms improved the ability to absorb protocatechuic acid in the small intestine and the susceptibility to metabolization by the microbiome [25].

### 3.2. Ascorbic Acid, Carotenoids, and Other Antioxidants

A characteristic feature of *Cydonia* and *Chaenomeles* fruits is the high content of vitamin C (ascorbic + dehydroascorbic acids) compared to the more common fruits of the Rosaceae family, such as apples, pears, or plums. The study by Souci et al. [77] determined 13 mg/100 g fm, which is only slightly more than in apples, while Sharma et al. [58] found a slightly higher value, i.e., 17 mg/100 g fm.

The literature shows higher vitamin C content in *Chaenomeles* than in *C. oblonga* fruits and significantly higher than in other common fruits [78]. Vila et al. [71] found 18–50 mg per 100 mL of *Chaenomeles* juice obtained from fruits harvested in the southern growing areas where its increased production was observed. Hellín et al. [69] obtained 45–78.5 mg of ascorbic acid in 100 mL of *C. japonica* juice, but significantly more in *C. speciosa*, *C. cathayensis*, and *C. × superba* fruit juices (102, 103, and 109 mg/100 mL, respectively). Bieniasz et al. [79] found it in a wide range of 68–207 mg/100 g fm depending on genotype and season, Hallmann et al. [80] measured it at 63 mg/100 g fm, while Zhang et al. [78] obtained values in a similar range of 69–159 mg/100 g. In turn, Baranowska-Bosiacka et al. [16] confirmed that fruits contain a substantial amount of ascorbic acid (55–92 mg/100 g fm) and this content remains relatively stable during storage and processing. Mezhenskij [81] found that fresh *C. × superba* fruits contain 60–150 mg of this acid per 100 g, based on data collected over eight years. The values obtained by Hallmann et al. [80] and mentioned above were only about twice lower than those of fruits considered to be unique sources of this vitamin, i.e., wild rose (*Rosa rugosa* Thunb.), and about 60% lower than those of rowan berries (*Sorbus aucuparia* L.).

The fruits of *Cydonia* contain carotenoids, which are antioxidants known to quench reactive oxygen species, including very harmful singlet oxygen. Souci et al. [77] determined 0.05 mg of carotene in 100 g fm and 5.5 µg of its derivative, retinol (vitamin A). The fruit material also contained thiamine (vitamin B_1_, 30 μg/100 g), riboflavin (vitamin B_2_, 30 µg/100 g), and niacin (vitamin B_3_, 0.2 mg/100 g), but not biotin and folic acid as found in an apple. Legua et al. [82] showed that the total concentration of carotenoids was higher in the peel (0.16–0.86 mg/100 g, depending on the clone) than in the pulp (0.04–0.42 mg/100 g) and that the color of the peel did not correlate with the color of the pulp. In recent studies by Najman et al. [83,84], the authors compared the total *trans* carotenoid content in fresh, dried, and processed fruits and obtained higher values. The β-carotene content was 13.6 mg/100 g fm, and the xanthophyll levels were significantly lower: 3.5 and 1.4 mg/100 g fm for lutein and zeaxanthin, respectively. Drying the fruit at 50 °C and 70 °C, freeze-drying, cooking, and frying increased the content of zeaxanthin and β-carotene by about five times. Lutein was more sensitive to conventional drying, but all types of processing also contributed to the increase in this xanthophyll.

A paper by Hallmann et al. [80] showed that among the carotenoids, *C. japonica* fruits contained mainly lutein (40 µg/g fm), lycopene (20.5 μg/g fm), and a small amount of β-carotene (1.7 µg/g fm). In a study by Turkiewicz et al. [85], *Chaenomeles* fruits of three species, i.e., *C. × superba*, *C. japonica*, and *C. speciosa*, and 19 cultivars contained 32–315 mg/kg dm of carotenoids (and some cultivars of *C. × superba* were the richest in carotenoids), 5.5–38 mg/kg dm of tocopherols, and 2–42 mg/kg dm of tocotrienols (both groups of vitamin E activity). Five carotenoids (all-*trans*-lutein, all-*trans*-β-cryptoxanthin, all-*trans*-α-carotene, all-*trans*-β-carotene, and 9- or 9′-*cis*-β-carotene), as well as four isomers of tocopherols and four tocotrienols, were identified in the fruits tested, regardless of cultivar. The predominant carotenoid was β-carotene and the predominant tocopherol was α-tocopherol, making these fruits a valuable source of provitamin A and vitamin E. Subsequent investigations by Turkiewicz et al. [75] have shown that *Chaenomeles* leaves can also be a good material for obtaining a tocopherol-rich extract whose content values ranged from 0.7 to 10.7 IU depending on the cultivar (100 g dm of leaves cover on average 24% of the daily requirement for vitamin E). On the other hand, the product obtained from *C. japonica* seeds, i.e., cold-pressed seed oil, contained the highest amounts of tocopherols (726 mg/kg) and β-carotene (11 mg/kg) compared to sesame, poppy, peanut, flaxseed, pumpkin, sunflower, almond, hazelnut, and walnut oils [33].

Among the compounds with proven biological effects, including antioxidant activity, pentacyclic triterpenes also play an essential role. Ursolic and oleanolic acids are characteristic chemical markers of *Chaenomeles*, which can be used to evaluate and classify the quality of this plant [86]. The presence of a new acylated triterpene (3-(*O*-(*E*)-3,5-dihydroxycinnamoylursolic acid) together with ursolic, oleanolic, and pomolic acids was demonstrated by Xu et al. [87].

### 3.3. Minerals

*C. oblonga* fruits are rich in mineral elements, especially Ca, K, and P, making them almost twice as rich in minerals as an apple [1,29]. Other studies, however, showed average amounts compared to the most commonly consumed fruits in Europe, i.e., K: 248 mg/100 g; P: 26 mg/100 g; Na: 8 mg/100 g; and Ca: 18 mg/100 g [57].

*C. japonica* fruits are also rich in minerals compared to other Rosaceae fruits, especially Fe and Mo, making them one of the richest sources of these elements. Also noteworthy are the high contents of Mg, Na, Cu, Zn, and P [3,88], although these contents were similar to those determined in *C. oblonga* fruits. The analysis by Baranowska-Bosiacka et al. [16] confirmed the high content of micro (Fe, Cu, Zn, Mn, and Mo) and macro (Mg, Ca, P, K, and Na) elements. The contents of Fe and Mo in these tests were 0.516 mg and 0.02 mg per 100 g dm, respectively. There are significant differences in the content of individual minerals in the fruits of related genera: *Chaenemeles* and *Pseudocydonia* are also interesting. For example, the content of K in the fruits of *C. japonica* was 249 mg/100 g, in *C. speciosa* it was much lower (up to 147 mg/100 g), and in *P. sinensis* K was not detected at all [88]. A study by Hellín et al. [69] found similar concentrations of K, ranging from 153 mg (*C. cathayensis*) to 241 mg (*C. speciosa*) in 100 mL of juice. *C. japonica* fruits were the most abundant in Mg, as confirmed by Hellín et al. [69], while *P*. *sinensis* contained the highest amounts of Fe and Mn (2.6 and up to 3.1 mg/100 g, respectively). The contents of Cu, Zn, and Ca were similar in all fruits of these species [78,88,89].

### 3.4. Carboxylic Acids

The fruits of all the species discussed in this review owe their distinctive flavor to their high content of organic acids. The presence of citric, malic, and fumaric acids has been confirmed in both the peel and pulp of *Cydonia* fruits [35,44]. Of these, citric acid was the most quantitatively determined, followed by malic and oxalic acids [44]. According to Silva et al. [54], in quince pulp and peel, the sum of malic and quinic acids represented 93%, so all other acids were present in minimal amounts, less than 0.5%, with the exception of citric and ascorbic acids. The sum of all quantified acids was about 7 g/kg in both pulp and peel, which is in agreement with the previously reported results [35].

There is a limited amount of research on the carboxylic acid content of *Chaenomeles* fruits. It is known that the fruits of *C. × superba* contain 4–5% organic acids (data collected over eight years) [81]. In a study by Hellín et al. [69], three acids (malic, quinic, and succinic) were detected in the fruit juices of five *Chaenomeles* taxa: *C. japonica*, *C. speciosa*, *C. cathayensis*, *C. japonica* × *C. speciosa*, and *C. × superba*. The typical organic acids such as citric, oxalic, tartaric, and galacturonic acids were not found in detectable amounts. The concentration of malic acid was similar in all tested juices, ranging from 3.06 to 5.09 g/100 mL. Slightly more significant differences were observed in the case of quinic acid, the juice from *C. speciosa* fruit containing significantly more of it. The same juice was particularly rich in succinic acid (174 mg/100 mL). In comparison, the other juices contained no more than 27.1 mg/100 mL (*C. japonica* and *C. japonica* × *C. speciosa*) and 52.5 mg/100 mL (*C. cathayensis*). Notably, Baranowska-Bosiacka et al. [16] showed a low oxalate content (8.21 mg/100 g fm) in the fruits of *C. japonica*.

### 3.5. Carbohydrates Including Fiber

Analyses by Lesińska et al. [90] showed that fresh *C. oblonga* fruits contained 7.18% of sugars, while Sharma et al. [58] determined 9% of total sugars, including 5% of reducing sugars in the juice. According to Rasheed et al. [91], 100 g of pulp contained 13.4 g of carbohydrates, of which 5.15 g was reducing sugars. HPLC analyses revealed the presence of monosaccharides: rhamnose, mannose, glucose, arabinose, and galactose [92,93]. Lesińska et al. [90] indicated that fructose was the dominant sugar (61.6%), followed by glucose, which accounted for 22.4%. The authors also showed that the total sugar content in *C. oblonga* was lower than in apples, pears, plums, and cherries.

*Chaenomeles* fruits contained about twice as much sugar as *Cydonia* fruits (3.8% bm) [90]. Nine carbohydrates were identified in their juice, i.e., stachyose, raffinose, sucrose, glucose, xylose, rhamnose, fructose, inositol, and sorbitol [69]. The dominant sugar was fructose, followed by glucose [69,90]. Considering the sugar content in fruit juices of different taxa, *C. cathayensis* is noteworthy as it contained 2–3 times more glucose and about twice more fructose than other juices tested [69].

The fruit of *C. oblonga* is known for its high pectin content, which makes it suitable for use in the food industry as a gelling ingredient, and for its crude fiber, which is beneficial to the digestive system, alleviating gastrointestinal disorders and cardiovascular diseases, and inhibiting the formation of some gastrointestinal cancers [1,91]. The average content of pectin in fruits of different varieties was 2 g/100 g [1] or 1.8 g/100 g [58]. For crude fiber, it ranged from 1.56 to 1.65 g/100 g [90]. Similar values of 1.6% and 1.9% were found by Sharma et al. [58] and Hegedus et al. [94].

Studies on fiber in *Chaenomeles* fruit have yielded more inconsistent results. Thomas et al. [95] distinguished three groups of quince genotypes: a low-fiber group (three genotypes, 28–30 g/100 g dm), a medium-fiber group (nine genotypes, 30–36 g/100 g dm), and an isolated genotype (*Chaenomeles speciosa*) that contained a considerable amount of fiber (38 g/100 g dry matter). Studies on cell wall polysaccharides showed that 100 g of dry fruit contained 11 g of pectins, 3 g of hemicelluloses, and 18 g of cellulose residues [96]. Later research by Thomas et al. [97] confirmed the above-mentioned pectin contents in *C. japonica* fruits, i.e., 11 g per 100 g dm and 1.4 g per 100 g fm. Hellín et al. [98] showed the high content of dietary fibers and pectins in *C. japonica* fruits, which encouraged them to use the juice to improve the quality of bread. *P. sinensis* fruits have been shown by Qin et al. to contain a high amount of polysaccharides, accounting for 11% of the dry pulp [99]. According to the authors, this fruit can be used as a source of commercial pectin due to its high pectin content. On the other hand, Baranowska-Bosiacka et al. [16] found only 4.7% dietary fiber in fresh *C. japonica* fruit. Similarly, a study by Mezhenskij [81] showed that the fruits of *C. × superba* contained a lower amount of pectins and significantly less than those of *C. oblonga*, i.e., only 0.6%. These differences were probably due to the different maturity of the fruits. The highest pectin content was found in unripe fruits.

## 4. Biological Activity of Quince Fruits

The fruits of *C. oblonga* have been used since ancient times in the Middle East and the Mediterranean region. It is an essential plant in Iranian traditional medicine (ITM) and modern phytotherapy, which is used to prevent or treat many diseases such as cancer, diabetes, hepatitis, ulcers, and respiratory and urinary tract infections [93,100]. In this review, I have focused on the properties of the fruits, but a literature analysis shows that most of the works describing the health-promoting properties of *C. oblonga* concern the leaves [101]. The dried fruits of *Chaenomeles* are one of the most important drugs in traditional Chinese medicine (TCM). They have been used for thousands of years to treat asthma, colds, sore throats, tuberculosis, mastitis, and hepatitis [102]. In TCM, *C. speciosa* fruit is used to treat gastric disorders, dyspepsia, dysentery, enteritis, influenza, and rheumatic inflammation [103,104]. *P. sinensis* fruit has been used alone or in combination with other herbs to treat diarrhea, vomiting, muscle aches, and colds [105], as well as an antitussive, antiflatulent, and diuretic. It is also known for its expectorant activity [106] and its extract is traditionally used to treat viral infections [107].

**Figure 4:**
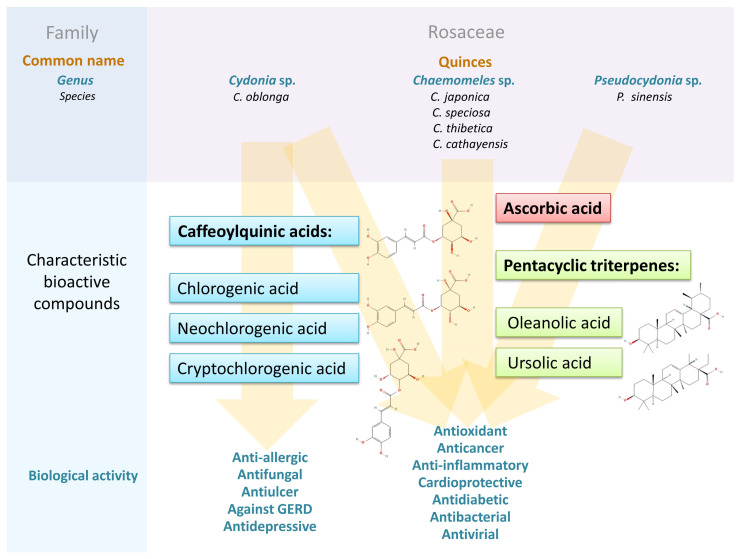
The characteristic bioactive compounds and biological effects of quince (*C. oblonga*, *Chaenomeles* sp., and *P. sinsnsis*) fruits [108].

In this review, the biological activities of the analyzed fruits, described in the available literature, have been segregated and presented below, taking into account the dominant ones, and realizing that due to the complexity of the molecular mechanisms leading to the disorder development, this could be prepared and discussed in many ways. The relevant data are extracted in Table 5 and summarized in Figure 4 [108].

### 4.1. Antioxidant Properties

In general, antioxidant activity is attributed to radical scavenging, prevention of chain reaction initiation, binding of transition metal ion catalysts, decomposition of peroxides, and prevention of continuous hydrogen uptake [159]. In addition to cell-produced antioxidant enzymes and low-molecular-weight antioxidants such as Cys or glutathione (GSH), valuable components of the human diet are plant antioxidants derived from a large group of secondary metabolites that may be helpful in the treatment of diseases associated with the overproduction of reactive oxygen and nitrogen species (ROS/RNS). Numerous studies have shown that low-molecular-weight antioxidants may play an important role in the prevention and treatment of many human diseases, including those resulting from a highly processed diet, the presence of environmental pollutants, and inappropriate lifestyle choices [160,161]. Therefore, the various effects of food isolates, including those from quince, may play a cocktail role in therapies whose common features are oxidative stress and inflammation. The topic of using quince fruit extracts appears most often in the literature due to their significant content of antioxidants [36,37,53,61,111,162,163,164,165,166], including polyphenols and ascorbic acid. However, it is worth mentioning that these fruits also contain other potent antioxidants such as carotenoids, tocopherols, and tocotrienols, as well as minerals (including Fe and Mn) that are cofactors of antioxidant enzymes.

Numerous studies have demonstrated that *C. oblonga* tissues are rich in phenolic acids and flavonoids, which are potent antioxidants [36,37,38,39,41,43,164,167]. Phenolics can act as antioxidants in several ways: as reducing agents, hydrogen donors, free radical scavengers, and singlet oxygen quenchers [158]. One of the first studies using TBARS (thiobarbituric acid reactive substances) and ABTS^+^ (3-ethyl-benzothiazoline-6-sulfonate) assays showed that its fruits were among the 5 fruits with the highest antioxidant/antiradical capacity out of 28 tested [111]. Scavenging activities against DPPH (2,2-diphenyl-1-picrylhydrazyl) and peroxyl radicals as well as protective activities against erythrocyte damage were observed by Costa et al. [46]. They showed significantly greater reducing power than that of green tea, which is often used as a reference plant, but the antioxidant properties varied significantly depending on the extraction method and extractant. Torres and colleagues [56] analyzed the antiradical capacities of *C. oblonga* and apple fruits using DPPH and ORAC (oxygen radical absorbance capacity) assays. The results showed that quince had 40% higher scavenging DPPH radicals expressed in Trolox equivalents and 50% higher ORAC values, respectively, than apple.

In turn, Baroni et al. [109] analyzed acidified extracts from *C. oblonga* pulp and jam made from the pulp. They obtained high values of scavenging DPPH radicals (2166 µM Trolox/100 g fresh pulp) and FRAP (ferric ion reducing antioxidant power; 2433 µM Trolox/100 g fresh pulp). The processing of quince did not significantly affect the antioxidant capacity as measured by the above two tests, since it was 60% of the starting material. In their subsequent work, extracts obtained similarly to the above were evaluated in terms of the effect of processing and simulated digestion on the antioxidant properties of quince jam. Oral digestion showed that only 30% of its phenolics were bioavailable. After gastric digestion, this percentage increased to 44%. In turn, after digestion and absorption in the small intestine, only 2.7% and 24% of the original phenolics were detected in the dialyzed and non-dialyzed fractions, respectively. Quinic acids were found to be the most resistant to digestion [55].

Yildirim et al. [112] analyzed the antioxidant activity and reducing power of aqueous, ethanolic, and ethereal extracts of *C. oblonga* leaves and showed that the latter had the highest total antioxidant activity although it had low reducing power. On the other hand, the ethanolic extracts had the highest reducing power while the ethereal extracts had the lowest. Methanol leaf extract obtained before fruit ripening has also been successfully used in studies to alleviate hematotoxic stress induced by UV-A radiation [44].

In a study by Pacifico et al. [113], aqueous fermented *C. oblonga* fruit extract effectively scavenged DPPH and the anion superoxide radical with *ID*_50_ values of 69 µg/mL and 74 µg/mL, respectively. In contrast, quince lipophylic wax extract was more effective in preventing the formation of thiobarbituric acid reactive species (TBARS) with an *ID*_50_ of 49 µg/mL.

Scientific data on the antioxidant properties of *Chaenomeles* plants are less documented. One of the older works [115] showed that powder processed from *C. speciosa* showed good scavenging activity against DPPH, with a scavenging rate of 945 µg/g and 700 U/mL, and a FRAP value of 173 µmol Fe^2+^/g. In the work by Du et al. [59], the antioxidant activity of the extracts was investigated by ABTS^+^, FRAP, and DPPH assays. The highest value of Trolox equivalent antioxidant capacity (TEAC) was obtained for *C. speciosa*, which was 310 and 97 µmol/g fm with ABTS^+^ and FRAP, respectively, while *C. thibetica* extract was slightly less effective, exhibiting TEAC values of 254 and 84 µmol/g fm with ABTS^+^ and FRAP, respectively. It is worth noting that the fruits of both species showed more significant antioxidant properties than goji (*Lycium ruthenicum* Murray) and guava. *C. japonica* extracts had the lowest TEAC values (118 and 19 µmol/g with ABTS^+^ and FRAP, respectively). *P. sinensis* fruit extract was also the most effective in scavenging the DPPH radicals, followed by *C. speciosa*, while *C. japonica* extract was the least effective. Among the five extracts, the values obtained for four (excluding *C. japonica*) were between those of standard antioxidants such as ascorbic acid and BHT but were higher than those of Trolox [62]. Pearson correlation analysis confirmed that polyphenols, including proanthocyanins, are potent antioxidant and radical scavenging compounds in quince extracts [62,168]. The work by Baranowska-Bosiacka et al. [16] showed a significant content of antioxidants in *C. japonica* fruits and demonstrated that its aqueous extract had a hepatoprotective effect, observed as a decrease in the concentration of lipid peroxides. According to a recent study [72], the juice and pomace exhibited radical scavenging capacities of 15 and 70 µmol TE/100 g and 152 and 938 µmol TE/100 g, respectively, as measured by DPPH and ABTS^+^.

A study by Zhang et al. [123] showed that 2 of the 13 components isolated from *C. speciosa* fruits, i.e., 3,4-dihydroxybenzoic acid and quercetin, exhibited the highest DPPH scavenging activity, with *IC*_50_ values of 1.02 and 3.82 μg/mL, respectively. On the other hand, Deng et al. [116] found two peptides (RHAKF and NNRYE) in *C. speciosa* seeds after protein hydrolysis. RHAKF has been shown to scavenge DPPH radicals and superoxide anions, inhibit lipid peroxidation, and, in addition, inhibit tyrosinase [114], which may be an ingredient in cosmetics due to its involvement in melanogenesis. In terms of cosmetological applications, a partially similar potential use was found for *P. sinensis*. The sarcocarp extract was characterized by an effect similar to superoxide dismutase (SOD) and a pronounced collagenase inhibitory activity. The extract contained condensed tannins as the main polyphenolic components [169]. A type IV collagenase inhibitory effect was also observed for *C. japonica* fruit extract [131]. According to the authors, the main bioactive constituents were proanthocyanidins. There are several reports in the literature on the antioxidative properties of various products and by-products obtained from quince, e.g., polysaccharides from *P. sinensis* seed meal, which is a by-product of oil processing, used as fertilizer and animal feed [170]. Ma et al. [117] demonstrated that a 70% ethanolic extract of *C. thiberica* fruit, which is rich in phenolic compounds and has in vitro antioxidant activity, increased the levels of catalase (CAT), SOD, and GSH in rats. Additionally, the extract reduced MDA, a product of free-radical-induced oxidation of unsaturated fatty acids, which is treated as an indicator of lipid peroxidation. It also showed a protective effect on rats with chronic liver injury injected with CCl_4_ via the mitogen-activated protein kinase/nuclear factor (erythroid-derived 2)-like2 (MAPK/Nrf2) pathway.

### 4.2. Anti-Inflammatory, Anti-Allergic, and Various Immunomodulatory Effects

Recent studies on plant secondary metabolites give hope for the development of naturally derived drugs that could contribute to the treatment of diseases associated with chronic inflammation, such as rheumatoid arthritis, gastritis, inflammatory bowel disease, atherosclerosis, cancer, and many others [110].

Kawahara and Iizuka [119] evaluated the effect of a crude hot water extract of *C. oblonga* fruit on IgE-dependent late-phase immune responses of mast cells using an in vitro system. The extract reduced the induction of intracellular cyclooxygenase (COX)-2 expression but not COX-1 expression in mouse bone marrow-derived mast cells. It also reduced the elevation of interleukin (IL)-13 and tumor necrosis factor (TNF)-α expression levels and suppressed these cytokine expressions as well as leukotriene C_4_ and prostaglandin D_2_ production in the cells tested [3,119]. 

*C. oblonga* hot water extract was also found to have an inhibitory effect on type I allergies by suppressing immunoglobulin E (IgE) production and IgE-mediated degranulation [120]. NC/Nga mice fed with the extract showed a significant decrease in the development of atopic dermatitis-like skin lesions under conventional conditions. Serum IgE concentration was reduced in a dose-dependent manner, and the release of β-hexosaminidase from the rat basophilic leukemia cell line RBL-2H3 was inhibited. The extract fraction with masses below 3 kDa reduced the mRNA expression of the high-affinity subunit of the IgE receptor γ (Fc″RI) [120].

A combination of lemon juice and aqueous *C. oblonga* extract (Gencydo^®^) is traditionally used in anthroposophical medicine for the treatment of allergic rhinitis and asthma by down-regulating soluble mediators that are essential for the initiation of allergic reactions. It has been proven that Gencydo^®^ reduced the degranulation and histamine release of IgE-activated basophils and mast cells and inhibited the increase in IL-8, TNF-α, and granulocyte–macrophage colony-stimulating factor (GM-CSF) production in mast cells. In addition, it partially blocked eotaxin release from human bronchial epithelial cells, but did not affect the viability and activation of GM-CSF-activated eosinophil granulocytes [121].

Although the anti-inflammatory activity of *C. oblonga* fruits has been confirmed in many studies, the mechanisms of action of individual compounds often remain unclear. Recent studies using network pharmacology proved to be useful in the prediction of the anti-inflammatory mechanism of the quinic acids in which the *C. oblonga* fruit is extraordinarily abundant, namely chlorogenic, neochlorogenic, and cryptochlorogenic acids, as well as their four metabolites. They demonstrated anti-inflammatory effects through 52 common targets. Their analysis indicated that a chlorogenic acid homolog and its metabolites could act as signal molecules binding to these targets and regulating the biological functions of related targets. Enrichment analysis showed that the top ten pathways were p75 (NTR)-mediated signaling, MAPK-related pathways, glutathione conjugation, S1P2 pathway, TNF receptor signaling pathway, p38 MAPK signaling pathway, ALK1 pathway, Phase II conjugation, biological oxidations, and dopamine degradation [171].

An ethanolic extract of *P. sinensis* fruit, long used as a folk medicine for cough, revealed significant inhibitory effects on the pruritogenic compound 48/80 calcium oxalate monohydrate (COM)-induced scratching behavior in mice. Quercetin, catechin, and apigenin derivatives (apigenin-7-glucronide and apigenin-9-methoxy-7-glucronide, first found in *P. sinensis* fruits) showed significant inhibitory effects on COM-induced scratching behavior. The active fraction and these compounds also inhibited scratching induced by serotonin, platelet-activating factor, and prostaglandin E_2_, confirming that *P. sinensis* fruit can relieve itching in allergic patients [106].

As many reports have shown, there are potent antioxidants among the polyphenols of *C. oblonga* peel extract. In addition, a study by Essafi-Benkhadir et al. [110] also showed its anti-inflammatory properties, inhibiting TNF-α and IL-8 in a dose-dependent manner and increasing the levels of the anti-inflammatory IL-10 secreted by lipopolysaccharide (LPS)-treated macrophages. Analyses showed that this extract inhibited LPS-mediated activation of three major cellular pro-inflammatory effectors: nuclear factor-κB (NF-κB), p38MAPK, and protein kinase B (Akt).

The group of Li [124] found that chlorogenic acid was one of the components responsible for the anti-inflammatory effect of *C. speciosa,* which is confirmed by the results presented above [122]. Its 10% ethanolic fraction showed significant anti-inflammatory effects in the xylene-induced ear edema test, the acetic-acid-induced peritoneal capillary permeability test, and the cotton pellet granuloma test in mice or rats; it also showed marked analgesic activity in the acetic acid-induced abdominal contraction test and the formalin-induced paw licking test in mice and rats. Chlorogenic acid has also been found in the fruits of other *Chaenomeles* taxa and, as mentioned above, in large amounts in the pulp of *C. oblonga*.

Three compounds isolated from the ethanolic extract of *C. speciosa*, namely 3,4-dihydroxybenzoic acid, quercetin, and methyl-3-hydroxybutanedioic acid ester, were found to inhibit the production of TNF-α in RAW264.7 macrophage leukemia virus-transformed cells. In addition, quercetin was found to be active in the release of IL-6 with an inhibition rate of 39.8% [125]. The studies performed on the whole ethanol extract of *C. speciosa* showed significant inhibition of the activity of both COX-1 and COX-2, but the extract was more than twice as active against COX-2 as against COX-1 [125].

In general, *C. speciosa* has long been used as an herbal medicine for the treatment of various inflammatory diseases such as rheumatoid arthritis, prosopalgia, and hepatitis. Several pentacyclic triterpenoids, such as oleanolic, ursolic, betulinic, and maslinic acids, are known for their anti-inflammatory properties [126,172]. Recent work by Fallon et al. [173] demonstrated that plant pentacyclic triterpenes, including oleanolic acid, can modulate the expression of the nuclear bile acid receptor, farnesoid X receptor (FXR), which is a regulator of several intestinal functions. FXR activation reduces the production of pro-inflammatory cytokines, thereby contributing to reduced epithelial permeability. Highlighting the importance of the discovery, the authors proposed introducing a common name describing the new functional class of triterpenes as “FXR-targeted” nutraceuticals.

The glucoside fraction from *C. speciosa* was found to exert an anti-inflammatory effect in the collagen-induced arthritis rat model by suppressing the inflammatory response and restoring the body weight and immune organ weight of the rats. These glucosides also reduced lymphocyte proliferation and IL-1, IL-2, and TNF-α production in peritoneal macrophages and synoviocytes [126]. Additional studies have also confirmed that *C. speciosa* glycosides have antinociceptive effects related to their inhibitory effects on peripheral inflammatory mediators. The glycosides were shown to reduce the levels of PGE2 and TNF-α in the synovial cells of rats with adjuvant arthritis [127]. Recent research [128] showed that *C. speciosa* polysaccharides suppressed the secretion of pro-inflammatory cytokines (TNF-α and IL-1β) and COX-2, as well as the phosphorylation of c-Jun N-terminal kinase (JNK) and extracellular signal-regulated kinase (ERK1/2) in LPS-stimulated cells. Thus, the secretion of pro-inflammatory cytokines and the downregulation of MAPK signaling promoted the analgesic and anti-arthritic effects of *C. speciosa* polysaccharides [128].

The anti-inflammatory activity of *C. japonica* in lipopolysaccharide (LPS)-activated murine macrophages was also investigated using a polyphenol-rich leaf extract. The studies confirmed its involvement in reducing the expression of pro-inflammatory cytokines (IL-1β, IL-6, and TNF-α), inflammatory mediators (COX-2 and iNOS), and both NF-κB p65 and p-NF-κB p65 in LPS-stimulated cells [122].

The inhibitory effects of *P. sinensis* fruit extract on the inflammatory response of human mast cells were investigated by Kim et al. [107]. The authors found that the fruit extract inhibited the migration of human mast cell line (HMC-1) cells, which play an important role in various inflammatory diseases, in response to stem cell factor (SCF). The extract also inhibited TNF-α expression by blocking the activation of ERK, p38MAPK, and JNK in HMC-1 cells. It also suppressed the expression of IL-6, IL-8, and monocyte chemoattractant *protein*-1 (MCP-1) in human monocytic THP-1 cells as well as the secretion of IL-6 in human keratinocytic HaCaT cells.

### 4.3. Anticancer Activity

A study by Carvalho et al. [38] showed that *C. oblonga* fruit and leaf extracts exhibited significant antiproliferative activities, showing concentration-dependent growth inhibitory activity against human colon cancer cells (*IC*_50_ = 239.7 for 43.2 μg/mL), while no effect was observed in renal adenocarcinoma cells. The authors suggested that chlorogenic acid, present in all tested extracts of *C. oblonga* fruits and leaves, was responsible for this effect. The aqueous extract of fermented fruits, in addition to its antioxidant properties mentioned above, also exerted various proliferative and cytotoxic effects on several human cancer cell lines, such as HepG2, A549, and HeLa [38].

Riahi-Chebbi et al. [129] demonstrated that a polyphenolic extract from the peel of *C. oblonga* induced proliferation arrest and apoptosis of LS174 colon cancer cells and that such an effect was at least partially mediated by the inhibition of NF-κB activation. The extract also reduced the expression and secretion of VEGF-A by tumor cells, which could lead to the inhibition of tumor-induced angiogenesis. The authors attributed the observed effect to numerous polyphenols in the extract. According to Adiban et al. [130], an aqueous extract of *C. oblonga* fruit reduced serum biomarkers of liver damage in rats with hepatocellular carcinoma, including α-fetoprotein (AFP), γ-glutamyl transpeptidase (GGT), ALT, and AST. In addition, the extract showed antioxidant activity in vivo, increasing GSH levels and preventing lipid peroxidation in liver tissue.

The studies showed that water-soluble polysaccharides extracted from *C. speciosa* inhibited sarcoma 180 tumor growth in mice in a dose-dependent manner. Additionally, they increased the relative spleen index and body weight, concanavalin A- and lipopolysaccharide-induced splenocyte proliferation, and peritoneal macrophage phagocytosis. In addition, treatment with water-soluble polysaccharides could alleviate delayed-type hypersensitivity and promote the secretion of IL-2, TNF-α, and IFN-γ in the serum of tumor-bearing mice [174]. *C. speciosa* fruits, being rich in pentacyclic triterpenoids, have long been the subject of research using cancer cells. Several of them, including ursolic, oleanolic, and maslinic acids, typical for *Chaenomeles*, exhibit significant anticancer effects through the modulation of a diverse range of molecular targets and signaling pathways to induce cell cycle arrest and apoptosis as well as inhibiting cancer cell proliferation, progression, angiogenesis, tissue invasion, and metastasis [175].

Recent studies have demonstrated numerous anticancer properties of ursolic acid, particularly in breast cancer, hepatocellular carcinoma, cervical cancer, lung cancer, melanoma, gallbladder cancer, and prostate cancer [176]. On the other hand, oleanolic acid has been used in the treatment of various cancer cell lines, such as MCF-7 and MCF-7/ADR human breast cancer cells, the 1321N1 astrocytoma cell line, hepatocellular carcinoma, and HCT-116 colorectal cancer cells [177]. The third of the most widely distributed *C. speciosa* triterpenoids, maslinic acid, also showed pronounced inhibitory effects against various cancer cell lines, including stomach, pancreatic, and human colon cancer cells [178].

Procyanidin extract from *C. japonica* fruit influenced the activity of matrix metalloproteinases (MMP-2 and MMP-9) secreted into the culture medium by human peripheral blood mononuclear cells and by human leukemia HL-60, which may make these condensed tannins promising chemopreventive agents [131]. A flavanol-rich preparation from *C. japonica* fruit induced favorable changes in the Bax/Bcl-2 mRNA ratio, making normal and cancer cells more resistant and sensitive to apoptosis, respectively. The most favorable Bax/Bcl-2 ratio was found in DU145 human prostate cancer cells. The growth and invasiveness of MDA-MB-231 human breast cancer cells were strongly inhibited by the *C. japonica* preparation. This was accompanied by a reduction in MMP-9 activity and stimulation of tissue inhibitor of metalloproteinases, TIMP-1, expression (MMP-9/TIMP-1 ratio is an indicator in the assessment of invasion and metastasis) [132]. A similar flavanol preparation from the fruit of *C. japonica*, rich in mono- and oligomers of procyanidins, inhibited the expression of COX-2, MMP-9, and NF-κB, suggesting that it has cytotoxic, anti-inflammatory, and antiproliferative effects against SW-480 colon cancer cells [133]. In a study by Zvikas et al. [134], sixteen phenolics were detected in *C. japonica* leaves, with chlorogenic acid being the predominant compound. Incubation with the extracts reduced the viability of HROG36 glioblastoma cells with an efficiency similar to that of temozolomide, a drug used to treat glioblastoma. In the case of C6 glioblastoma cells, the extracts were even more effective than temozolomide.

Although studies on the anticancer properties of *C. japonica* have mainly been conducted on the fruit, there are also reports on the leaves. Crude phenolic leaf extract and purified phenolic-rich extracts contained 33 and 36 phenolics, respectively, of which chlorogenic acid and naringenin hexoside were found to be the major components. FRAP and ABTS^+^ tests showed that the purified phenolic-rich extract had two times higher antioxidant activity and exhibited higher cytotoxic activity against colon cancer cells (SW-480 and HT-29) than the crude phenolic extract. In addition, the purified phenolic-rich extracts had more potent cytotoxic effects on the colon cancer cell lines (SW-480 and HT-29) than on normal intestinal cells [135].

Gao et al. [136] investigated the anticancer activity of 22 functional constituents (including triterpenoids, flavonoids, and lignans) isolated from *P. sinensis* against human anaplastic large cell lymphoma (JB6) cells. This primary screening selected several compounds with promising anticancer activity.

Natural products of plant origin, including extracts rich in polyphenols, are able to alter cell signaling through epigenetic changes, including DNA methylation and histone modifications. These changes lead to altered expression of microRNA (miRNA) which are involved in the regulation of gene expression post-transcriptionally. A single miRNA is able to target more than one hundred genes. Moreover, each gene contains multiple binding sides for miRNAs. The possibility of developing a phytotherapeutic approach based on miRNAs isolated from medicinal plants may be the next step toward new-generation therapies. So far, the pharmaceutical industry has focused on plant secondary metabolites, excluding the concept of exploring potential biological functions at the expense of exogenous miRNAs [179]. However, the research confirms that they are involved in multiple signaling pathways, including the deeply studied cancer regulation processes, especially cell proliferation, and migration, metastasis, apoptosis, and cell differentiation [180]. Among the species tested, only the *C. oblonga* genome was analyzed for miRNAs. By using a trained SVM classifier, the identification of 600 putative pre-miRNA coding loci was carried out. Subsequent homology searches identified 33 matches, including 28 pre-miRNAs from *M. domestaina*, two from *Glycine max*, two from *Vitis vinifera*, and one pre-miRNA from *Paeonia lactiflora* [181]. As many studies have confirmed, miRNA can be regulated by many compounds obtained from medicinal plants. They are also found in *C. oblonga* fruits: e.g., caffeic acid, which can affect breast cancer and hepatocarcinoma cells, catechin, influencing lung and prostate cancers, hepatoma, and neuroblastoma, and quercetin, which is believed to have an effect on lung and pancreatic cancer cells [180]. Oleanolic acid, which is a valuable component of *Chaenomeles* and *Pseudocydonia* fruits, has been found to exhibit antiproliferative potential by upregulating tumor suppressor miR-122 both in in vitro and in vivo models of lung carcinoma [182]. Unfortunately, the genomes of the last-mentioned genera are poorly studied.

### 4.4. Cardioprotective Effects

Early prevention of hyperlipidemia is an important factor in reducing the incidence of cerebral and cardiovascular diseases. It has been shown that total flavonoid extracts from *C. oblonga* fruits and leaves could regulate blood lipid metabolism in rats by scavenging oxygen free radicals and improving antioxidant potential [138]. Working on hyperlipidemic rats, the authors showed that total flavonoids from the extract significantly reduced the concentration of total cholesterol, triglycerides, and low-density lipoprotein cholesterol (LDL cholesterol), and increased high-density lipoprotein cholesterol (HDL cholesterol) in serum.

*C. oblonga* leaf extracts are used in TCM to treat or prevent cardiovascular disease. This type of *C. oblonga* activity has been experimentally supported by research by Abliz et al. [137], Zhou et al. [139,140,141], and Abulizi et al. [142]. In the studies by Abliz et al. [137], ethanolic leaf extract reduced total cholesterol, triglycerides, LDL-cholesterol, and MDA, inhibited the activity of aminotransferase (ALT), aspartate aminotransferase (AST), and lipopolysaccharides, while it increased the HDL-cholesterol content and the activity of SOD, glutathione peroxidase (GSH-Px), lipoprotein lipase (LPL), and hepatic lipase (HL) in the serum of hyperlipidemic rats fed with the extract. The total flavonoid preparations of *C. oblonga* fruits and leaves were also effective in reducing ALT and AST, showing their involvement in hepatocyte protection. They also improved the activity of SOD and GSH-Px in liver tissues, which inhibited the formation of MDA [138]. On the other hand, Abulizi et al. [142] investigated the possibility of using aqueous extracts of *C. oblonga* in the treatment of atherosclerosis. They concluded that the extracts could reduce the degree of aortic injury and hemodynamic indices, regulate blood lipid levels, and improve liver function in rats with atherosclerosis. The authors also observed an increased activity of SOD and GSH-Px and a decreased content of MDA in the serum of atherosclerotic rats. Furthermore, they specified the active compounds among the 14 identified in the extract and the mechanisms underlying the anti-atherosclerotic effects of the extract using a molecular docking approach.

Hypertensive disease, its causes, and numerous consequences, including those on the functioning of the circulatory system, are widely discussed in the literature. The magnitude of this problem is still underestimated, while, according to projections, in 2025 there will be 1.5 billion people living with hypertension in the world [183]. By feeding *C. oblonga* ethanolic extracts to renal hypertensive rats, Zhou et al. [139] selected a dose that produced similar effects to captopril, a drug used to treat essential or renovascular hypertension. The extracts significantly reduced whole blood viscosity and improved erythrocyte deformability. Subsequent attempts to use *C. oblonga* extract by Zhou et al. [140] concerned its effect on renal hypertension, which is a common cause of secondary hypertension in humans, usually as a result of renal artery stenosis or hypertrophy. The study analyzed the dose–response effect of ethanolic leaf extracts on hypertension and biomarkers related to blood pressure control. It was observed that it lowered the concentration of peptides: angiotensin II (AII), one of the most effective blood pressure regulators, which causes intense contraction of the muscles of small blood vessels and significantly increases blood pressure, thus accelerating the heart rate, as well as endothelin (ET). The authors concluded that these extracts have properties similar to those of angiotensin-converting enzyme (ACE) inhibitors and captopril. Subsequently, Zhou et al. [141] demonstrated the antithrombotic activity of an aqueous extract of *C. oblonga* leaves in mice and rats, probably at least partly related to an antithromboxane effect. Their results were compared with those of acetylsalicylic acid and showed that the quince extracts dose-dependently prolonged the thrombosis occlusion time, reduced the weight of arterial and venous thrombosis, decreased the plasma concentrations of thromboxane B_2_, and increased that of 6-keto-prostaglandin F1α. As suggested by the authors, their effect on the prostacyclin/thromboxane balance was probably beneficial in their antithrombotic activity.

*C. oblonga* extract has been shown to be effective in alleviating cardiotoxicity caused by the use of a popular drug, doxorubicin (DOX), which is effective in the treatment of various types of cancer. It has been suggested that mitochondria play a critical role in these mechanisms of toxicity. A study by Gholami et al. [143] showed that *C. oblonga* fruit ameliorated the impairment of cardiac mitochondrial function in DOX-treated rats by preventing mitochondrial ROS generation, lipid peroxidation, swelling, membrane potential decrease (%ΔΨm), and cytochrome *c* release, and also by increasing mitochondrial GSH and complex II activity. Hanan et al. [144] performed an in vivo study to evaluate DOX-induced cardiotoxicity. Rats were orally administered quince peel extracts at doses of 160 and 320 mg/kg bm for 30 days, and ECG analysis was performed at the end of the experiment. In addition, lipid profile, blood serum parameters (creatine kinase MB (CK-MB), LDH, and AST), and tissue parameters (MDA, SOD, GSH, CAT) were analyzed. The groups of pretreated rats significantly attenuated DOX-induced changes in all parameters. In addition, improvement in histopathologic changes in cardiac tissue was also observed in the pretreated groups, indicating regression of cardiac injury.

Regrettably, there is limited research on this subject with regard to other species, as outlined in this review. *C. speciosa* powder dietary supplement at concentrations of 5 and 10% was administered to mice and significantly reduced serum low-density lipoprotein cholesterol and total cholesterol levels. A significant increase in GSH-Px activity and total antioxidant capacity and a decrease in the relative atherosclerotic plaque area of the aortic sinus and arch were observed compared to the control group [115]. A triterpenoid 28-*O*-β-*D*-glucopyranosyl-2α,3β-dihydroxyolean-12-ene-24,28-dioic acid, named chaenomelosid A, and its aglycone, chaenomelogenin A, isolated for the first time from the fruit of *P. sinensis*, showed tissue tromboplastin (TF) inhibitory activity, which may be helpful in the regulation of blood coagulation [145].

### 4.5. Antidiabetic Activity

The increasing incidence of diabetes mellitus is alarming and is becoming one of the most significant health problems worldwide, mainly associated with hyperglycemia and abnormal lipid and antioxidant profiles [118]. In addition to the involvement of antioxidant and anti-inflammatory compounds present in *C. oblonga* extract in the reduction of factors contributing to the development of ischemic heart disease, their activity in the treatment of type II diabetes has been widely described. Polysaccharides from *C. oblonga* fruit have also been found to inhibit tyrosine phosphatase activity (*IC*_50_ for 2.07 µg/mL), indicating its ability to treat type II diabetes and obesity [93,112]. In the in vitro study by Tang et al. [146], *C. oblonga* seed extract stimulated glucose metabolism by activating the PI3K/AKT insulin signaling pathway in L6 myotubes. Recent studies have shown promising results regarding the use of *C. oblonga* fruit extract and suggest that it can be used as an anti-obesity agent [147] by reducing body weight, body fat mass, and serum insulin, triglyceride, and leptin concentrations. However, it increased serum adiponectin and HDL cholesterol levels in high-fat diet-induced C57BL/6 mice. The extract increased AMPK activation and inhibited adipogenesis [147].

There are several papers showing the antidiabetic effect of *C. oblonga* leaves. They are used as a folk remedy for the treatment of this disease in Turkey. Oral administration of hydroethanolic extracts (500 mg/kg) for 5 days to diabetic rats reduced blood glucose levels by 34%. It induced a significant antioxidant effect on heart tissue as measured by TBARS concentration. The observed effects were more pronounced than those obtained with Jerusalem artichoke (*Helianthus tuberosus* L.) tuber and leek (*Allium porrum* L.) bulb extracts [148]. The same effect as at doses of 250 mg/kg and 500 mg/kg was observed during the use of an antidiabetic drug (tolbutamide) at a dose of 100 mg/kg. According to recent work by He et al. [184], the therapeutic effect of chlorogenic acid isoforms contained in *Pyrrosia petiolosa* (Christ) Ching, a traditional Chinese medicine used in the treatment of diabetes with good effectiveness, is realized by promoting insulin secretion and pancreatic tissue repair, which results in a strong hypoglycemic effect.

Based on recent data, Zakłos-Szyda and Pawlik [149] concluded that *C. japonica* polyphenols could be suitable for the prevention of pre-diabetes, type II diabetes, and metabolic syndrome. Here, *C. japonica* polyphenolic extract was tested on glucose metabolism in a human hepatoma HepG2 cell line cultured under non-metabolically altered and hyperglycemic conditions. Pretreatment with the preparation caused a decrease in intracellular ROS generation and affected mitochondrial membrane polarization, which appeared to lead to AMP-activated protein kinase (AMPK) activation. Other effects observed in HepG2 cells were associated with an increase in glucose uptake and glycogen content as well as alleviation of gluconeogenesis through modulation of PEPCK, PTP1B, enzymes, FOXO1 transcription factor, and glucose transporter GLUT2/4 expression.

However, a more recent paper by Loza-Rodríguez et al. [185] showed that not only the polyphenol fraction can contribute to the regulation of glycemia, but also one of the most common triterpenoids of the *Chaenomeles* genus, i.e., oleanic acid. In myoblasts, oleanolic acid increased peroxisome proliferator-activated receptors γ/α (PPARγ/α) expression of mRNA and their regulated genes. Protein expression of PPARγ, GLUT4, and fatty acid transport protein 1 (FATP1) was also increased, and GLUT4 translocation was observed.

The group of Sancheti [150] found that the constituents of *P. sinensis* fruit are an effective glycosidase inhibitor. The crude 80% methanolic extract and its fractions were tested for α- and β-glucosidase and α- and β-galactosidase inhibitory activities. The results concluded that this fruit contains α-glucosidase and β-glucosidase inhibitors and can be used as a powerful natural drug in the treatment of type II diabetes by controlling glucose absorption. Subsequent studies by this group showed that oral administration of *P. sinensis* extract (500 mg/kg bm) significantly inhibited the progression of streptozotocin (STZ)-induced diabetes in rats, and this effect may be related to its hypoglycemic effect, modulation of lipid metabolism, and ability to scavenge free radicals. The authors observed increased liver glycogen content, SOD, GSH, and CAT levels, and decreased fasting blood glucose, blood urea nitrogen, serum total cholesterol, triglycerides, LDL cholesterol, ALT, and AST concentrations [118]. Using the ethyl acetate fraction of *P. sinensis* extracts, Sancheti et al. [151] demonstrated an ameliorative effect on impaired blood glucose, lipid, acetylcholinesterase, and antioxidant levels in STZ-induced diabetic rats. According to the authors, these effects could be mediated via the inhibition of glucose transporter, α- and β-glucosidase, amylase, and lipase, and its significant antioxidant potential.

### 4.6. Antiviral and Antibacterial Activity

The use of neuraminidase (NA) inhibitors is one of the most common approaches in the development of anti-influenza drugs. Three compounds isolated from the ethanolic extract of *C. speciosa*, namely 3,4-dihydroxybenzoic acid, methyl-3-hydroxybutanedioic acid ester, and vomifoliol, exhibited significant dose-dependent inhibition of NA activity with *IC*_50_ values of 1.27, 1.90, and 2.33 μg/mL, respectively. The studies also showed that most of the 13 compounds isolated from the extract inhibited the production of NO (which can exacerbate lung injury after influenza virus pneumonia) by more than 25% at 5 μg/mL in RAW264.7 cells [123].

Several studies have shown that the anti-influenza effect of fruits and vegetables depends on the presence of certain polyphenols, and the mechanisms of inhibition vary depending on the molecular structure. The antiviral role of *P. sinensis* fruit polyphenols is appreciated in TCM, but so far poorly documented in the scientific literature [107,186,187]. Pretreatment with a polyphenol-rich *P. sinensis* extract was shown to slightly reduce cell binding, hemagglutination, and hemolytic activity in influenza A-infected Madin–Darby canine kidney epithelial cells, as well as the synthesis of viral cRNA, vRNA, and secondary mRNA [187]. High-molecular-weight polyphenols from *P. sinensis* fruit have also been shown to neutralize influenza virus by inhibiting heme agglutination activity and suppressing influenza NS2 protein synthesis [186].

*C. oblonga* peel extract was found to be the most active in inhibiting bacterial and yeast growth (Gram-positive *Staphylococcus aureus* and Gram-negative *Pseudomonas aeruginosa*, somewhat less so in the cases of *Escherichia coli* and yeast *Candida albicans*) with minimum inhibitory and bactericidal concentrations in the range of 10^2^ × 10^3^–10^5^ × 10^3^ mg polyphenol/mL [37].

A study by Alizadeh et al. [152] showed that the extract of *C. oblonga* can be helpful against diarrhea and in controlling Enterobacteriaceae infections; the ethanolic seed extract was the most effective against *E. coli*, while the aqueous fruit extract showed an antimicrobial effect only on *Escherichia aerogenes.* The crude extract of polyphenols from *C. oblonga* fruits showed antibacterial activity against *E. coli*. Five polyphenols were isolated and tested for their activity, namely 5-*O*-caffeoylquinic acid, quinic acid, a derivative of quinic acid, proanthocyanin B_1_, and methyl 5-*O*-caffeoylquinate, revealing the strong inhibitory properties of quinic acid and its derivative [188].

Interestingly, *C. oblonga* fruit extract has been found to be an effective agent in supporting the treatment of COVID-19. It was recommended by the Indian Ministry of AYUSH, Government of India, as an ingredient in a mixture against the SARS-CoV-2 virus and was described as an antioxidant, immunomodulatory, anti-allergic, smooth muscle relaxant, and anti-influenza agent [153].

Urbanavičiūtė et al. [72] demonstrated various antibacterial properties of *C. japonica* fruits. Strong inhibition of the growth of Gram-positive bacteria (*E. faccalis*, *B. subtilis,* and *S. aureus*) was observed, which, thanks to the analysis of three cultivars, was correlated with a high content of rutin and epicatechin. *E. coli* and *P. aeruginosa* were inhibited to a lesser extent. However, the activity of the extract against the fungus *C. albicans*, observed in tests with *C. oblonga* extracts, was not observed here at all. The antimicrobial activity of *C. speciosa* extract was evaluated against 18 Gram-negative or Gram-positive bacteria and a fungal strain and showed that the extract had a better inhibitory effect on *S. aureus*, *S. typhimurium*, MRSA, *E. coli*, *P. aeruginosa*, *S. epidermidis*, *Y. enterocolitica*, and *C. albicans* than ampicillin sodium salt, fluconazole, and berberine chloride. The better inhibitory effect of *C. speciosa* extract was especially noticeable for drug-resistant bacteria [72]. Another study highlighted differences in the antibacterial activity of 23 main compounds of this fruit against *S. aureus* and *E. coli* [103].

Procyanidins, which are considered one of the main fractions of *C. oblonga* polyphenols and found in great diversity in *Chaenomeles*, are believed to modulate the intestinal microbiome. They act as inhibitors of digestive enzymes and show a direct, strong antimicrobial effect. Furthermore, the health benefits of dietary fiber in plants are believed to depend on its interactions with phytochemicals, including tannins [189].

### 4.7. Other Health-Promoting Properties

In Persian medicine, heated extract of *C. oblonga* has been used to treat gastroesophageal reflux disease (GERD), the most common esophageal disorder. It is defined as the reflux of stomach contents into the esophagus with troublesome symptoms such as heartburn, vomiting, abdominal pain, recurrent pneumonia, and erosive esophagitis. In the work of Zohalinezhad et al. [156], *C. oblonga* syrup was helpful in the treatment of GERD in pediatrics as a gastric tonic and ulcer healing agent. Quince syrup significantly improved the patient’s condition and symptomatic status and was effective for at least two weeks after drug administration. Shakeri et al. [155] administered concentrated *C. oblonga* fruit extract, also known as “quince sauce”, to 137 pregnant women with GERD and found that the efficacy of quince sauce for the treatment of pregnancy-related GERD was similar to that of the popular drug ranitidine. Later, a randomized, double-blind clinical trial was conducted in 96 children with suspected GERD, and the results showed that the administration of quince syrup was also helpful in improving GERD in children, and its efficacy was similar to that of ranitidine [154].

Pretreatment of rats with *P. sinensis* jelly from boiling water fruit extracts protected gastric tissues against HCl/ethanol-induced gastric injury, as evidenced by a reduction in gastric lesion index and TNF-α levels. The authors suggested that the pronounced gastroprotective activity of *P. sinensis* jelly may be due to the synergistic effect of components such as pectin and highly polymerized procyanidins, which have a strong binding affinity to the gastric mucosa [190].

Biologically active compounds from *C. oblonga* have been shown to be helpful in the treatment of gynecological disorders. A randomized, triple-blind, controlled clinical trial of 146 women with menorrhagia showed that the *C. oblonga* pill was as effective in reducing menstrual bleeding and increasing hemoglobin levels as mefenamic acid, a nonsteroidal anti-inflammatory drug (NSAID) known for its side effects [157]. *C. oblonga* syrup was found to be significantly more effective against nausea and vomiting during pregnancy than vitamin B_6_, which is often used to relieve morning sickness. The beneficial effects of quince were even more significant because the treatment was characterized by high safety [191].

In turn, research by Din Ganaie et al. [158] demonstrated the antidepressant effects of aqueous and ethanolic extracts of *C. oblonga* fruit in rats using the forced swim test (FST) and tail suspension test (TST), which were compared with the effects of treatment with a standard drug (imipramine). In addition, antidepressant activity was confirmed by a gradual increase in serum enzymatic antioxidant levels. Co-administration of *C. oblonga* with imipramine has been introduced as a novel therapeutic approach in the treatment of depression.

## 5. Conclusions

The review presented here revealed an extensive literature on the bioactive compound contents and uses of fruits of *Cydonia oblonga*, *Chaenomeles* sp., and *Pseudocydonia sinensis*.

*Cydonia oblonga* fruit is relatively well-known and appreciated culinarily. Its anti-inflammatory, immunomodulatory, and anticancer properties, as well as its protective effect against disorders of lipid and carbohydrate metabolism, and thus of the cardiovascular system, are attributed to the presence of numerous polyphenols, including protocyanidins and caffeoylquinic acids. Their use in the treatment of GERD and allergies seems unique. The analysis of the literature showed that most of the papers describing the health-promoting properties of *C. oblonga* focused on leaves.

There are fewer studies on the medical and culinary uses of the genus *Chaenomeles*, but *C. speciosa* seems to be the most studied for its potential medicinal applications. In addition to its potent anti-inflammatory effects, the fruits have been characterized for their ability to lower glycemia and improve lipid metabolism. In turn, *C. japonica* is the most valuable additive that improves the taste of food products. The fruits of *Chaenomeles* seem to be unappreciated in terms of their use.

*P. sinensis* fruit is an ingredient of many medicines used in TCM. There is quite a bit of research on the biological effects of its fruits, mainly immunomodulatory and antidiabetic, but much of the work is old and inaccessible. Unfortunately, despite its beneficial health value, the plant seems to have a more local significance.

## 6. Future Perspectives

A comparison of the properties of the fruits of the three genera shows that the potential of *Chaenomeles* sp. and *Pseudocydonia* fruits is still poorly recognized. Overall, they contain more phenolic compounds and ascorbic acid than *Cydonia* fruits and are rich in carotenoids and tocopherols, organic acids, and minerals (Fe and Mo) as well as triterpenes with proven anticancer activity. The chemical characteristics of these fruits open the way to the potential use of their extracts or individual compounds in pharmacology as an alternative to conventionally used drugs. The sensory characteristics of *Chaenomeles* sp., largely related to the high content of organic acids, are unique and indicate that these fruits could be used on a larger scale. Due to their remarkable antioxidant properties, more significant than those of *C. oblonga* fruits, they could be used as natural preservatives in the food and pharmaceutical industries. In view of these advantages, it should be concluded that *Chaenomeles* sp. fruits are valuable functional food ingredients; however, little is known about the molecular action of their phytochemicals. Progressive pharmacology such as miRNA therapies may be the next best step towards identifying new therapeutic options using these medicinal plants. Future research efforts should delve deeper into the systematic investigation of potentially active secondary metabolites and biological effects exerted by fruit extracts and, in parallel, undertake species-specific genomic analysis.

## Figures and Tables

**Table 1:** Phenolic contents in *C. oblonga* fresh and dried pulp.

Pulp Compound	Main Extractant	Content		Ref.
3-CQA (3-*O*-caffeoylquinic acid) neochlorogenic acid	Acetone	5.68	mg/100 g fm	[37]
		10.89	mg/100 g fm	[39]
	Methanol	68.46	mg/100 g dm	[36]
		50.00	mg/100 g fm	[38]
		100.00	mg/100 g fm	[41]
		252.00	mg/100 g fm	[52]
	Water:acetone	14.10	mg/100 g fm	[53]
	Water:methanol	2.87	mg/100 g fm	[35]
		9.46	mg/100 g fm	[54]
4-CQA (4-*O*-caffeoylquinic acid) cryptochlorogenic acid	Acetone	2.29	mg/100 g fm	[39]
	Acidified methanol	7.97	mg/100 g dm	[36]
		3.01	mg/100 g fm	[55]
	Water:acetone	1.50	mg/100 g fm	[53]
	Water:methanol	0.47	mg/100 g fm	[35]
		0.85	mg/100 g fm	[54]
5-CQA (5-*O*-caffeoylquinic acid) chlorogenic acid	Acetone	15.57	mg/100 g fm	[37]
		15.72	mg/100 g fm	[39]
	Acidified methanol	12.83	mg/100 g fm	[55]
	Methanol	64.88	mg/100 g dm	[36]
		67.00	mg/100 g fm	[38]
		142.00	mg/100 g fm	[41]
	Water:acetone	12.30	mg/100 g fm	[53]
	Water:ethanol	14.53	mg/100 g fm	[40]
	Water:methanol	8.54	mg/100 g fm	[35]
		9.00	mg/100 g fm	[54]
3,5-di-CQA (3,5-di-*O*-caffeoylquinic acid)	Acetone	13.96	mg/100 g fm	[39]
	Methanol	5.63	mg/100 g dm	[36]
		7.00	mg/100 g fm	[41]
	Water:methanol	0.53	mg/100 g fm	[35]
		0.93	mg/100 g fm	[54]
3-*p*-Coumaroylquinic acid	Acidified methanol	0.44	mg/100 g fm	[55]
5-*p*-Coumaroylquinic acid	Acidified methanol	2.27	mg/100 g fm	[55]
*p*-Coumaroylquinic acid	Acetone	1.21	mg/100 g fm	[39]
(+)-Catechin	Acetone	7.20	mg/100 g fm	[37]
	Acidified methanol	0.06	mg/100 g fm	[55]
(−)-Catechin	Acetone	0.18	mg/100 g fm	[37]
Catechin	Water:ethanol	0.02	mg/100 g fm	[40]
(−)-Epicatechin	Acidified methanol	2.25	mg/100 g fm	[55]
Epicatechin	Water:ethanol	0.11	mg/100 g fm	[40]
Kaempferol rutinoside	Acidified methanol	0.08	mg/100 g fm	[55]
Kaempferol hexoside	Acidified methanol	0.38	mg/100 g fm	[55]
Phloridzin	Water:ethanol	<0.01	mg/100 g fm	[40]
Procyanidin B_1_	Methanol	2.00	mg/100 g fm	[52]
	Water:ethanol	6.52	mg/100 g fm	[40]
Procyanidin B_2_	Water:acetone	1.40	mg/100 g fm	[53]
Quercetin	Acidified methanol	0.37	mg/100 g fm	[55]
Q-3-Gal (quercetin-3-*O*-galactoside) hyperin	Acidified methanol	5.64	mg/100 g fm	[55]
	Water:methanol	0.06	mg/100 g fm	[35]
		0.25	mg/100 g fm	[54]
Q-3-G (quercetin-3-*O*-glucoside)	Methanol	2.00	mg/100 g fm	[52]
Q-3-R (quercetin-3-*O*-rutinoside) rutin	Acetone	9.05	mg/100 g fm	[37]
		0.51	mg/100 g fm	[39]
	Acidified methanol	2.27	mg/100 g fm	[55]
	Methanol	3.30	mg/100 g dm	[36]
		2.00	mg/100g fm	[41]
		146.00	mg/100 g fm	[52]
	Water:ethanol	0.005	mg/100 g fm	[40]
	Water:methanol	0.53	mg/100 g fm	[35]
		0.33	mg/100 g fm	[54]

fm—fresh mass, dm—dry mass.

**Table 2:** Phenolic contents in *C. oblonga* fresh and dried peel.

Peel Compound	Main Extractant	Content		Ref.
3-CQA (3-*O*-caffeoylquinic acid) neochlorogenic acid	Acetone	3.94	mg/100 g fm	[37]
		26.46	mg/100 g fm	[39]
	Methanol	196.64	mg/100 g dm	[36]
		114.00	mg/100 g fm	[38]
		128.00	mg/100 g fm	[41]
	Water:methanol	5.55	mg/100 g fm	[35]
		20.99	mg/100 g fm	[54]
4-CQA (4-*O*-caffeoylquinic acid) cryptochlorogenic acid	Acetone	0.51	mg/100 g fm	[37]
		4.82	mg/100 g fm	[39]
	Methanol	17.44	mg/100 g dm	[36]
		18.00	mg/100 g fm	[38]
	Water:methanol	0.93	mg/100 g fm	[35]
		1.92	mg/100 g fm	[54]
5-CQA (5-*O*-caffeoylquinic acid) chlorogenic acid	Acetone	12.85	mg/100 g fm	[37]
		36.76	mg/100 g fm	[39]
	Methanol	182.94	mg/100 g dm	[36]
		165.00	mg/100 g fm	[38]
		184.00	mg/100 g fm	[41]
	Water:ethanol	41.13	mg/100 g fm	[40]
	Water:methanol	17.95	mg/100 g fm	[35]
		27.98	mg/100 g fm	[54]
3,5-di-CQA (3,5-*O*-dicaffeoylquinic acid)	Acetone	13.96	mg/100 g fm	[39]
	Methanol	9.87	mg/100 g dm	[36]
		13.00	mg/100 g fm	[41]
	Water:methanol	1.63	mg/100 g fm	[35]
		2.43	mg/100 g fm	[54]
(+)-Catechin	Acetone	5.07	mg/100 g fm	[37]
(–)-Catechin	Acetone	0.10	mg/100 g fm	[37]
Catechin	Acetone	3.40	mg/100 g fm	[39]
	Water:ethanol	0.20	mg/100 g fm	[40]
4-*p*-Coumaroylquinic acid	Water:ethanol	0.11	mg/100 g fm	[40]
*p*-Coumaroylquinic acid	Acetone	1.39	mg/100 g fm	[39]
Epicatechin	Water:ethanol	3.50	mg/100 g fm	[40]
Kaempferol	Acetone	12.60	mg/100 g fm	[40]
K-3-G (kaempferol-3-*O*-glucoside)	Acetone	10.65	mg/100 g fm	[37]
		2.48	mg/100 g fm	[39]
	Methanol	8.88	mg/100 g dm	[36]
		55.00	mg/100 g fm	[38]
		34.00	mg/100 g fm	[41]
	Water:methanol	3.54	mg/100 g fm	[35]
		2.58	mg/100 g fm	[54]
K-3-Gly (kaempferol-3-*O*-glycoside	Water:methanol	3.22	mg/100 g fm	[54]
	Methanol	14.00	mg/100 g fm	[38]
		25.00	mg/100 g fm	[41]
Kaempferol glycoside	Methanol	11.22	mg/100 g dm	[36]
Kaempferol glycoside acylated with *p*-coumaric acid A1	Water:methanol	1.84	mg/100 g fm	[54]
Kaempferol glycoside acylated with *p*-coumaric acid A1	Methanol	5.38	mg/100 g dm	[36]
Kaempferol glycoside acylated with *p*-coumaric acid A2	Water:methanol	3.45	mg/100 g fm	[54]
Kaempferol glycoside acylated with *p*-coumaric acid A2	Methanol	10.95	mg/100 g dm	[36]
Kaempferol glycoside acylated with *p*-coumaric acid	Methanol	24.00	mg/100 g fm	[38]
		5.00	mg/100 g fm	[41]
K-3-R (kaempferol-3-*O*-rutinoside)	Acetone	3.96	mg/100 g fm	[37]
		1.13	mg/100 g fm	[39]
	Methanol	15.22	mg/100 g dm	[36]
		2.10	mg/100 g fm	[41]
	Water:methanol	6.11	mg/100 g fm	[35]
		5.10	mg/100 g fm	[54]
Quercetin	Acetone	7.01	mg/100 g fm	[37]
Q-3-Gal (quercetin-3-*O*-galactoside) hyperin	Acetone	12.40	mg/100 g fm	[37]
		4.46	mg/100 g fm	[39]
	Methanol	49.12	mg/100 g dm	[36]
		329.00	mg/100 g fm	[38]
	Water:methanol	10.08	mg/100 g fm	[35]
		6.07	mg/100 g fm	[54]
Q-3-G (quercetin-3-*O*-glucoside) isoquercitrin	Acetone	9.23	mg/100 g fm	[37]
Quercetin glycoside acylated with *p*-coumaric acid A1	Methanol	16.69	mg/100 g dm	[36]
Quercetin glycoside acylated with *p*-coumaric acid A2	Methanol	6.57	mg/100 g dm	[36]
Quercetin glycoside acylated with *p*-coumaric acid	Acetone	5.92	mg/100 g fm	[37]
	Methanol	22.00	mg/100 g fm	[38]
		11.00	mg/100 g fm	[41]
	Water:methanol	5.20	mg/100 g fm	[54]
		1.77	mg/100 g fm	[54]
Quercetin glycosides acylated with *p*-coumaric acid	Methanol	4.00	mg/100 g fm	[41]
Q-3-R (quercetin-3-*O*-rutinoside) rutin	Acetone	47.21	mg/100 g fm	[37]
		17.59	mg/100 g fm	[39]
	Methanol	177.78	mg/100 g dm	[36]
		329.00	mg/100 g fm	[38]
	Water:ethanol	0.93	mg/100 g fm	[40]
	Water:methanol	51.73	mg/100 g fm	[35]
		74.08	mg/100 g fm	[54]
Phloridzin	Water:ethanol	>0.01	mg/100 g fm	[40]
Procyanidin B_1_	Water:ethanol	11.02	mg/100 g fm	[40]

fm—fresh mass, dm—dry mass.

**Table 3:** Phenolic contents in *C. oblonga* fresh and dried seed (extracted with methanol).

Seed Compound	Content		Ref.
3-CQA (3-*O*-caffeoylquinic acid) neochlorogenic acid	2.40	mg/100 g dm	[36]
	1.00	mg/100 g fm	[38]
	1.00	mg/100 g fm	[41]
4-CQA (4-*O*-caffeoylquinic acid) cryptochlorogenic acid	2.76	mg/100 g dm	[36]
5-CQA (5-*O*-caffeoylquinic acid) chlorogenic acid	5.44	mg/100 g dm	[36]
	6.00	mg/100 g fm	[38]
	5.00	mg/100 g fm	[41]
3,5-di-CQA (3,5-di-*O*-caffeoylquinic acid)	2.99	mg/100 g dm	[36]
6-*C*-Glucosyl-8-*C*-pentosyl chrysoeriol	1.61	mg/100 g dm	[36]
	5.00	mg/100 g fm	[38]
	6.00	mg/100 g fm	[41]
6-*C*-Pentosyl-8-*C*-glucosyl chrysoeriol	2.18	mg/100 g dm	[36]
	10.00	mg/100 g fm	[38]
	3.00	mg/100 g fm	[41]
Isoschaftoside	1.71	mg/100 g dm	[36]
	8.00	gm/100 g fm	[41]
Lucenin-2	1.02	mg/100 g dm	[36]
	3.00	mg/100 g fm	[38]
	3.00	mg/100 g fm	[41]
Schaftoside	1.14	mg/100 g dm	[36]
	5.00	mg/100 g fm	[38]
	6.00	mg/100 g fm	[41]
Stellarin-2	2.76	mg/100 g dm	[36]
	15.00	mg/100 g fm	[38]
	8.00	mg/100 g fm	[41]
Vicenin-2	1.46	mg/100 g dm	[36]
	7.00	mg/100 g fm	[38]

fm—fresh mass, dm—dry mass.

**Table 5:** Biological activities of quince (*C. oblonga*, *Chaenomeles* sp., and *P. sinensis*) fruits.

Activity	Species	Details	Ref.
Antioxidative	*C. oblonga*	prevention of hematotoxic stress	[44]
		in vitro effects	[46,55,56,109,110,111,112,113]
	*C. japonica*	in vitro effects	[16,59,62]
	*C. speciosa*	in vitro effects	[62,114]
		increase in GSH-Px activity and antioxidant capacity in mice serum	[115]
		in vitro activity of two peptides RHAKF and NNRYE	[116]
	*C. thiberica*	in vitro effects	[62]
		in vitro effects, increased CAT, SOD, and GSH content in rat serum	[117]
	*P. sinensis*	in vitro effects	[62]
		increase in SOD, GSH, and CAT levels in rat serum	[118]
Anti-inflammatory Immuno-modulatory	*C. oblonga*	inhibition of NF-κ98980B and p38MAPK, and Akt activation	[110]
		IgE-dependent late-phase immune reaction modulation in vitro	[119]
		suppressing IgE production in type I allergy	[120]
		various anti-histamine effects	[121]
	*C. japonica*	reduction in the expression of IL-1β, IL-6, TNF-α, COX-2, iNOS, NF-κB p65, and p-NF-κB p65 in RAW264.7 cells	[122]
	*C. speciosa*	inhibition of TNF-α production in RAW264.7 cells	[123]
		anti-inflammatory effects by standard tests in mice/rats	[124]
		inhibition of COX-1 and COX-2 activities	[125]
		reduction in lymphocyte proliferation, and IL-1, IL-2, and TNF-α production in peritoneal macrophages and synoviocytes	[126]
		reduction in PGE2 and TNF-α concentration in synoviocytes	[127]
		inhibition of TNF- α, IL-1β, and COX-2; JNK and ERK1/2 phosphorylation in NR8383 cells	[128]
	*P. sinensis*	inhibition of scratching induced by serotonin, platelet-activating factor, and prostaglandin E_2_	[106]
		inhibition of TNF-α expression by blocking ERK, p38(MAPK), and JNK activation in HMC-1 cells	[107]
Anticancer	*C. oblonga*	inhibitory activity toward human colon cancer cells	[38]
		cytotoxic effects on HepG2, A549, and HeLa cells	[113]
		apoptosis of colon cancer LS174 cells	[129]
		reduced liver damage in hepatocellular carcinoma	[130]
	*C. japonica*	activation of MMP-2 and MMP-9 secreted by leukemia HL-60 cells	[131]
		change in Bax/Bcl-2 ratio in DU145 prostate cancer cells; inhibition of MDA-MB-231 breast cancer cells	[132]
		COX-2 and MMP-9 inhibition, NF-κB expression, anti-metastatic activities towards SW-480 colon cancer cells	[133]
		reducing HROG36 glioma cell viability	[134]
		cytotoxic effect on SW-480 and HT-29 colon cancer cells	[135]
	*C. speciosa*	inhibition of sarcoma 180 cells by promoting secretion of IL-2, TNF-α, and IFN-γ in serum	[68]
	*C. thiberica*	protective effect on chronic hepatic damage *via* the MAPK/Nrf2 pathway	[117]
	*P. sinensis*	cytotoxic effect on human anaplastic large cell lymphoma JB6 cells	[136]
Cardio-protective	*C. oblonga*	lipid profile, blood serum parameter improvement	[137,138]
		essential and renovascular hypertension reduction	[139]
		renal hypertension reduction	[140]
		anti-thromboxane effect	[141]
		improving the degree of aortic injury and hemodynamic indicators	[142]
		DOX-induced cardiotoxicity alleviation	[143,144]
	*C. speciosa*	reduction in relative atherosclerotic plaque area of aortic sinus and aortic arch	[115]
	*P. sinensis*	increase in thromboplastin (TF) inhibitory activity	[145]
Antidiabetic	*C. oblonga*	inhibition of tyrosine phosphatase activity	[112]
		activating PI3K/AKT insulin signaling in vitro	[146]
		multifactorial anti-obesity effects	[147]
		blood glucose level reduction	[148]
	*C. japonica*	elevation of gluconeogenesis through modulation of PEPCK, PTP1B, FOXO1, and GLUT2/4 expression	[149]
	*P. sinensis*	hypoglycemic effect, modulation of lipid metabolism	[118]
		α- and β-galactosidase inhibitory activities	[150]
		inhibition of glucose transporter, α- and β-glucosidase, and amylase	[151]
Antibacterial Antiviral Antifungal	*C. oblonga*	against: *S. aureus*, *P. aeruginosa*, *E. coli*, and yeast *C. albicans*	[37]
		against *E. aerogenes* and *E. coli*	[152]
		against SARS-CoV-2 virus	[153]
	*C. japonica*	mainly against *E. faccalis*, *B. subtilis*, and *S. aureus*	[72]
Other	*C. oblonga*	treatment of gastroesophageal reflux (GARD)	[154,155,156]
		reducing menstrual bleeding and increasing hemoglobin levels	[157]
		antidepressant activity	[158]
